# Dietary carbohydrate restriction in type 2 diabetes mellitus and metabolic syndrome: time for a critical appraisal

**DOI:** 10.1186/1743-7075-5-9

**Published:** 2008-04-08

**Authors:** Anthony Accurso, Richard K Bernstein, Annika Dahlqvist, Boris Draznin, Richard D Feinman, Eugene J Fine, Amy Gleed, David B Jacobs, Gabriel Larson, Robert H Lustig, Anssi H Manninen, Samy I McFarlane, Katharine Morrison, Jørgen Vesti Nielsen, Uffe Ravnskov, Karl S Roth, Ricardo Silvestre, James R Sowers, Ralf Sundberg, Jeff S Volek, Eric C Westman, Richard J Wood, Jay Wortman, Mary C Vernon

**Affiliations:** 1 State University of New York Downstate Medical Center, Brooklyn, New York, USA; 2 New York Diabetes Center, Mamaroneck, New York, USA; 3 Private Practice, Njurunda, Sweden; 4 University of Colorado Health Sciences Center, Denver, Colorado, USA; 5 Albert Einstein College of Medicine, Bronx, New York, USA; 6 Division of Pediatric Endocrinology, University of California Medical Center, San Francisco, California, USA; 7 Manninen Nutraceuticals Oy, Oulu, Finland; 8 Ballochmyle Medical Group, Mauchline Ayrshire, UK; 9 County Hospital, Karlshamn, Sweden; 10 Independent Researcher, Lund, Sweden; 11 Department of Pediatrics, Creighton University, Omaha, Nebraska, USA; 12 Portuguese Sports Institute, Cruz Quebrada, Portugal; 13 Cosmopolitan International Diabetes Center, University of Missouri, Columbia, Missouri, USA; 14 Slottsstadens Läkarhus, Malmö, Sweden; 15 Department of Kinesiology, University of Connecticut, Storrs, Connecticut, USA; 16 Lifestyle Medicine Clinic, Duke University Medical Center, Durham, North Carolina, USA; 17 Springfield College, Springfield, Massachusetts, USA; 18 Health Canada, First Nations Division, Vancouver, British Columbia, Canada; 19 Private Practice, Lawrence, Kansas, USA

## Abstract

Current nutritional approaches to metabolic syndrome and type 2 diabetes generally rely on reductions in dietary fat. The success of such approaches has been limited and therapy more generally relies on pharmacology. The argument is made that a re-evaluation of the role of carbohydrate restriction, the historical and intuitive approach to the problem, may provide an alternative and possibly superior dietary strategy. The rationale is that carbohydrate restriction improves glycemic control and reduces insulin fluctuations which are primary targets. Experiments are summarized showing that carbohydrate-restricted diets are at least as effective for weight loss as low-fat diets and that substitution of fat for carbohydrate is generally beneficial for risk of cardiovascular disease. These beneficial effects of carbohydrate restriction do not require weight loss. Finally, the point is reiterated that carbohydrate restriction improves all of the features of metabolic syndrome.

## Background

The epidemic of diabetes continues unabated, and impassioned calls for better treatment and prevention strategies are common features of scientific conferences. While it is generally acknowledged that total dietary carbohydrate is the major factor in glycemic control, strategies based on reduction of dietary carbohydrate have received little support. The American Diabetes Association, for example, has traditionally recommend against low carbohydrate diets (less than 130 g/day[1]; while the most recent guidelines [2] admit such diets as an alternative approach to weight loss, they continue to emphasize concerns and downplay benefits. Similarly, the Diabetes and Nutrition Study Group of the European Association for the Study of Diabetes [3] reported "no justification for the recommendation of very low carbohydrate diets in persons with diabetes." We feel, however, that there is ample evidence to warrant an alternative perspective and that diets based on carbohydrate restriction should be re-evaluated in light of current understanding of the underlying biochemistry and available clinical data.

Whatever success low fat dietary approaches have had in improving diabetes is to be applauded but it is reasonable for patients to be aware of the potential benefits of an alternative approach which we present here. The key feature is that low carbohydrate diets are based on mechanism. That is, glucose directly or indirectly through insulin, is a major control element in gluconeogenesis, glycogen metabolism, lipolysis and lipogenesis. The downstream stimulus-response processes are a current research interest (see e.g. [4,5]) but, according to the view considered here, dietary fat has a generally passive role and deleterious effects of fat are almost always seen in the presence of high carbohydrate.

While low carbohydrate diets may not be appropriate for everyone, choices should be left to individual physicians and patients. Key points that bear on the assessment of benefit vs. risk of carbohydrate restriction are presented below. The discussion focuses on type 2 diabetes but many of the principles will apply to metabolic syndrome and possibly to type 1 as well[6,7].

1. Carbohydrate restriction improves glycemic control, the primary target of nutritional therapy and reduces insulin fluctuations.

2. Carbohydrate-restricted diets are at least as effective for weight loss as low-fat diets.

3. Substitution of fat for carbohydrate is generally beneficial for markers for and incidence of CVD.

4. Carbohydrate restriction improves the features of metabolic syndrome.

5. Beneficial effects of carbohydrate restriction do not require weight loss.

Carbohydrate restriction is an intuitive and rational approach to improvement of glycemic and metabolic control. Data demonstrating that weight loss and cardiovascular risk are also improved remove these barriers to the acceptance of carbohydrate restriction as a reasonable if not the preferred treatment for type 2 diabetes. Finally, carbohydrate restriction is a potentially favorable diet for improving components of the metabolic syndrome and thereby for the *prevention *of diabetes.

### 1. Carbohydrate restriction improves glycemic control, the primary target of nutritional therapy and reduces insulin fluctuations

**Figure 1:**
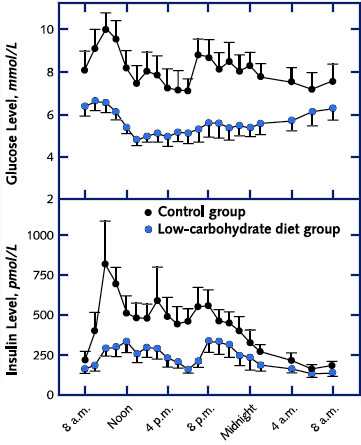
**Glucose and Insulin response for patients with type 2 diabetes on low carbohydrate diet vs. control**. Data (means ± SE) are for 9 patients with type 2 diabetes after seven days on their usual high-carbohydrate diet (control) and after 2 weeks) on a low-carbohydrate diet. Medication was reduced in 4 patients and discontinued in one during the low-carbohydrate diet. Figure redrawn from Boden, *et al*. [8].

Figure 1 shows glycemic and insulin responses in a carefully controlled inpatient comparison of 10 obese patients with type 2 diabetes[8]. Fourteen days of a low-carbohydrate diet led to a mean decrease in energy intake of approximately 1000 kcal/d, a reduction in plasma glucose levels and average hemoglobin A1c (HbA_1c_) from 7.3% to 6.8%. Insulin sensitivity improved by approximately 75%. No adverse effects were reported, and the carbohydrate that was removed was not replaced by substantial protein or fat.

Dashti, *et al*. showed dramatic and sustained reduction in blood glucose in 31 obese diabetic patients on a ketogenic diet over 56 weeks. Normal levels were reached by week 48 [9]. Similarly, Nielsen, *et al.*[10] reported that a 20% carbohydrate diet was superior to a 55–60% carbohydrate diet with regard to bodyweight, glycemic control and reduction in HbA_1c_. At follow-up, after 22 months, HbA_1c _remained improved. In a 16-week pilot study of Yancy, *et al*., 21 overweight participants with type 2 diabetes showed a mean decrease in HbA_1c _from 7.4% to 6.3%.

These results are not isolated. Many studies have demonstrated the benefits of carbohydrate reduction [11-16] on glycemic control. Reaven, Garg, Grundy and coworkers have shown benefits of even moderate carbohydrate reduction, from 55% to 40%[17,18].

#### Reduction or elimination of medication

A striking effect of carbohydrate restriction is reduction or elimination of medication. Table 1 shows results from Yancy, *et al*. [19] in which 17 of 21 patients with type 2 diabetes reduced or discontinued diabetes medication upon carbohydrate restriction. Similar results were found by Boden [8] and Nielsen [10,20]. Practitioners have pointed out the need to reduce medication in advance of undertaking a low carbohydrate diet [6,10,20,21] highlighting the power of carbohydrate restriction to bring about the same therapeutic effect as drugs.

**Table 1:** Changes in diabetes medication of 19 overweight participants with type 2 diabetes who underwent a 16-week diet intervention trial. Patients were provided with VLCKD counseling with an initial goal of <20 g carbohydrate/day. Medication was reduced at diet initiation. Data from Yancy, *et al*. [62].

**Patient number**	**Daily Dose – Week 0**	**Daily Dose – Week 16**
**Medications discontinued (n = 7 of 19 originally on medication)**		

5	glipizide 10 mg	none
	metformin 1000 mg	
6	metformin 1500 mg	none
7		none
9	metformin 1000 mg	none
15	metformin 1000 mg	none
22	metformin 1000 mg	none
24	metformin 1000 mg	none

**Medications reduced (n = 10 of 19)**		

3	70/30 insulin 50 units	metformin 1000 mg
	metformin 1000 mg	
11	metformin 2000 mg	metformin 2000 mg
	glyburide 20 mg	
16	metformin 2000 mg	metformin 2000 mg
	pioglitazone 45 mg	
	glypizide 20 mg	
21	metformin 1500 mg	metformin 1000 mg
	pioglitazone 30 mg	
8	NPH 145 units	NPH 25 units
	metformin 1000 mg	metformin 1000 mg
13	70/30 insulin 70 units	70/30 insulin 35 units
	metformin 2550 mg	metformin 2550 mg
23	70/30 insulin 110 units	70/30 insulin 80 units
	pioglitazone 45 mg	pioglitazone 45 mg
		metformin 1000 mg
25	NPH 70 units, r 30 units	NPH 8 units
	metformin 2000 mg	metformin 2000 mg
	pioglitazone 45 mg	pioglitazone 45 mg
27	70/30 insulin 86 units	70/30 insulin 18 units
	metformin 2000 mg	metformin 2000 mg
28	NPH insulin 90 units	NPH insulin 30 units
	lispro insulin 90 units	glypizide 20 mg
	glypizide 20 mg	

### 2. Carbohydrate-restricted diets are at least as effective for weight loss as low-fat diets

**Figure 2:**
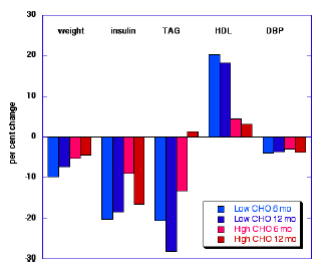
**Comparison of low and high carbohydrate diets at 6 and 12 months**. Results from a multi-center trial in which 63 obese men and women were randomly assigned to either diet. Data from Foster, *et al*. [26]. Figure from Volek & Feinman [24], used with permission.  DBP, diastolic blood pressure; TAG, triglycerides.

Low-carbohydrate diets generally perform better than low-fat diets for weight loss in normal subjects, and patients with metabolic syndrome or diabetes [22-25]. Studies by Foster, *et al*. [26] and Samaha, *et al*. [27] are often cited as examples where low carbohydrate diets are more effective at 6 months but no better than low-fat diets at 1 year. The experimental design, however, allowed re-introduction of carbohydrate in the low carbohydrate group as the study proceeded. Even if there were equal weight loss at one year, other physiologic markers, particularly TG and HDL, were greatly improved on the low-carbohydrate diet compared with the high carbohydrate diet as shown in Figure 2.

### 3. Substitution of fat for carbohydrate is generally beneficial for markers for and incidence of CVD

The diet-heart hypothesis states that dietary fat, or at least saturated fat, promotes CVD. There are, however, numerous counter-examples and the popular and scientific literature has seriously challenged many of the underlying assumptions of the hypothesis [28-33]. In fact, total fat in the diet is not associated with an increase in CVD, as shown by experiments going back to Ancel Keys's Seven Country Study [34]and, most recently and dramatically, the Women's Health Initiative [35].

#### Lipid markers for CVD

Clinically significant elevation of LDL-cholesterol is generally considered a primary indicator of CVD risk but interpretation must be tempered by the effect of particle size: small dense LDL particles are significantly more atherogenic than large, buoyant LDL particles [36,37]. Krauss, *et al*. identified a genetically influenced pattern (B) in people with higher levels of the smaller particles and found a strong linear relation between carbohydrate intake and prevalence of the atherogenic pattern B phenotype. Thus, replacing dietary fat with carbohydrate tends to worsen LDL size distribution for most of the population[36,37].

Other factors, such as high triglyceride (TG) and low HDL, are independent markers of insulin resistance and CVD risk. Indeed, the triglyceride:HDL ratio has been posited to be a surrogate measure of insulin resistance [38]. This ratio is frequently exacerbated under conditions that lower LDL [24]. An increase in apolipoprotein B (apoB) may be a preferred marker since each atherogenic lipoprotein particle contains one molecule of apoB; total LDL would bias results towards lower risk [39]. There is also strong evidence that the apoB/apoA-I ratio is superior to conventional cholesterol ratios [39] as a predictor of CVD risk. Of particular importance is circulating TG because of its mechanistic link to the formation of atherogenic particles [40,41], and its responsiveness to dietary manipulation. There is probably no dietary outcome as reliable as the reduction in TG due to carbohydrate restriction[41].

#### The role of saturated fat

A primary goal of current recommendations is to put limits on dietary saturated fat but published results are inconsistent (see e.g. [42]). Several critical reviews have pointed up the general failure to meet the kind of unambiguous outcomes that would justify blanket condemnation of saturated fat, *per se *[29,30,41,43,44]. Notably, during the obesity and diabetes epidemic, the proportion of dietary saturated fat decreased. In men, the *absolute *amount decreased by 14%. Similarly, the WHI revealed no difference in CVD incidence for people who consumed < 10% saturated fat or those whose consumption was > 14%[35]. Dreon, et al. [44] showed that increased saturated fat lead to a *decrease *in small, dense LDL. Perhaps most remarkable was a study by Mozaffarian [45] which showed that greater intake of saturated fat was associated with *reduced *progression of coronary atherosclerosis; greater carbohydrate intake was linked to increased progression.

In our view, inconsistencies in the experimental results with dietary saturated fat arise from a failure to distinguish between replacement by unsaturated fat or by carbohydrate [3]. In the former case, there is usually improvement in CVD risk or outcome (although it is not excluded that this is due to the effect of the unsaturated fat rather than reduction in the risk from the saturated fat). Replacement of saturated fat with carbohydrate, however, is almost always deleterious [46,47]. Again, the idea that carbohydrate is a control element determining the fate of ingested lipid is overriding.

The assumption that the dietary fatty acid profile is reflected in plasma distribution is not always true, especially for saturated fatty acids which seems to be subject to much metabolic processing [42]. It was also expected that an increase in total fat might show changes in lipid pattern but Raatz, et al. showed that such differences were extremely small between a low fat and high fat diet [48]. A recent report comparing two low-CHO groups that differed in dietary SFA showed little difference in plasma levels of stearic or palmitic acid [49]. Most telling, Volek's group compared a VLCK diet (% CHO:fat 12:59) with a low-fat (LF) diet (56:24) and found that after 12 weeks, SFA in TG and cholesteryl ester were lower in the VLCK group than the LF group even thought the low carbohydrate group had a 3-fold higher intake of dietary SFA [50].

### 4. Carbohydrate restriction improves the features of metabolic syndrome

An important idea guiding current medical thinking is that clustering of seemingly disparate physiologic states, obesity, atherogenic dyslipidemia, hyperglycemia and hypertension, termed metabolic syndrome (MetS) suggests a common underlying cause. Inherent in this concept lies the possibility that treating one risk factor or disease state might confer benefit for risk of other diseases. A recent review showed that carbohydrate restriction improves all of these markers[24]. Indeed, metabolic syndrome might be consistently defined as those physiologic markers that respond to reduction in dietary carbohydrate. Metabolic syndrome might be seen as a generalization of the carbohydrate intolerance that characterizes frank diabetes[24].

In a prospective study testing the hypothesis linking carbohydrate restriction to MetS, the carbohydrate-restricted group showed greater improvements in weight loss and multiple markers of atherogenic dyslipidemia (increased HDL and LDL diameter and reductions in TG and apo B/apo A1 ratio) compared to a low fat arm. Unexpectedly, the carbohydrate-restricted arm, with three times greater dietary saturated fat, showed a *reduction *in plasma saturated fat, while plasma saturated fat in the low-fat arm remained unchanged [50].

Similarly, Petersen, *et al*. [51] showed that ingestion of a high carbohydrate meal led to a greater increase in *de novo *fatty acid synthesis and hepatic triglyceride formation in insulin-resistant men compared to a similar group of insulin-sensitive controls. Carbohydrate-induced atherogenic dyslipidemia is thus enhanced by insulin resistance.

Carbohydrate restriction will generally reduce the consumption of fructose, which makes up half the mass of common sweeteners (high-fructose corn syrup or sucrose). Fructose consumption has been implicated in the epidemics of obesity, MetS, and type 2 diabetes and is known to induce hypertension, *de novo *lipogenesis, hepatic insulin resistance and adiposity [52-54].

In summary, carbohydrate restriction is one of the few common interventions that targets all of the features of MetS. If such a straight-forward approach can alleviate a condition for which there is no known effective drug, its potential should be vigorously explored.

### 5. Beneficial effects of carbohydrate restriction do not require weight loss

Obesity is commonly considered a cause of insulin resistance. Obesity, however, does not occur spontaneously. Obesity is a *response*. The effects of obesity that lead to insulin resistance in peripheral tissues, largely increased fatty acids, are downstream from the primary impact of diet. This argues for an emphasis of treatment on glycemic control and improved hepatic metabolism rather than weight loss. A simpler alternative hypothesis considers that insulin resistance represents a down-regulation of hormonal response as a result of persistent high levels of insulin, a feature common to other hormonal systems [55]. In this view, diabetes, obesity and the components of MetS are parallel effects of hyperinsulinemia and/or hyperglycemia.

**Figure 3:**
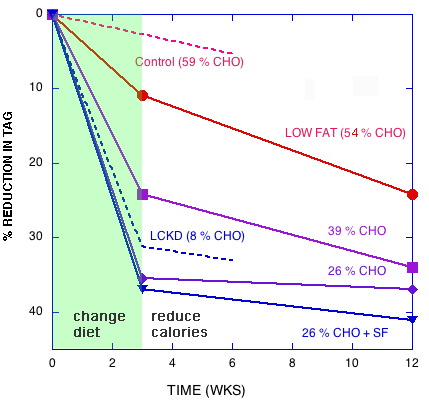
**Effect of dietary interventions on reduction in triglycerides**. Eucaloric diets of indicated carbohydrate content were begun at time 0. At week 3, a 1000 kcal reduction in energy was implemented and at week 9, dieters were put on maintenance diet. Combined effect of calorie reduction and maintenance are reported at week 12. **Solid Lines**: data from Krauss, *et al. *[58] were converted from reported log values in their Table 2 and per cent of baseline was calculated. **Dashed line**: data from Sharman, *et al *[56]: A eucaloric ketogenic diet was instituted for six weeks (no weight loss phase). Points were recorded at week 3 and 6. Figure modified from Feinman & Volek [57]. Similar results were found for HDL, apoB/apoA1 and other markers of CVD [57, 58]

The finding that lipid improvements seen in carbohydrate-restricted diets persist even after no further weight loss (Figure 2) suggests that the benefit of carbohydrate restriction is independent of weight loss. Two additional lines of evidence support this idea:

1) In experiments in which body mass is kept constant in normal-weight men[56]or patients with type 2 diabetes[11,13], a very low carbohydrate diet resulted in dramatic improvements in triglycerides and HDL cholesterol with minimal change in body mass.

2) Experiments in which change in macronutrients and weight loss are separated in time show that eucaloric carbohydrate reduction leads to greater improvement in atherogenic lipid markers (TG, HDL, apoB/apoA1 and mean LDL particle size) even in the presence of higher saturated fat[57,58]. A low fat diet, however, required weight loss to achieve effective improvement in the lipid profile (Figure 3). Notably, the sum of the two effects showed that eucaloric carbohydrate restriction plus weight loss was more effective than eucaloric low fat plus weight loss.

### Practical considerations and recommendations

#### Definitions and recommendations

Response to carbohydrate restriction shows both continuous, graded outcomes [17,18] as well as a threshold effects. LDL particle size, e.g. appears to depend linearly on the level of dietary carbohydrate[36,37]. On the other hand, many studies show maximum benefit for very low carbohydrate intake; the early phases of popular low carbohydrate diets target such very low levels [6,15,21,59,60]. The principle rests on the concept of a catalytic or threshold effect for insulin in shifting the body from an anabolic state to fat oxidation. The tipping point is empirically taken as the onset of ketonuria, also used as an indicator of compliance with a very low carbohydrate ketogenic diet (VLCKD). The threshold carbohydrate reduction for ketonuria varies among individuals, but a rough estimate is 50 g of carbohydrate per day or, approximately 10% of energy on a nominal 2000 kcal diet, (a target of 30 g/d is common in the early phases of popular VLCKD diets)[15,21,60].

We suggest the following definitions:

The ADA designates low carbohydrate diets as less than 130 g/d or 26% of a nominal 2000 kcal diet and we consider this a reasonable cutoff for the definition of a *low-carbohydrate diet*. Carbohydrate consumption before the epidemic of obesity averaged 43%, and we suggest 26% to 45% as the range for *moderate-carbohydrate diets*. The intake of less than 30 g/d, as noted above should be referred to as a *very low carbohydrate ketogenic diet (VLCKD)*. The term Ketogenic Diet should be reserved for the therapeutic approach to epilepsy. These diets do not independently specify the level of carbohydrate, but rather the sum of carbohydrate and protein.

In practice, many low carbohydrate dieters do not add additional fat. First shown by LaRosa, [61] it has now been observed by many other investigators. [8,62,63] A reduced carbohydrate diet may show significant per cent increase in fat, but there may be no change in the absolute amount consumed. Not everybody on a low carbohydrate diet follows this pattern, but a recommendation based on this behavior would seem more appropriate than unqualified rejection of low-carbohydrate diets.

While some proponents of carbohydrate restriction for the management of diabetes favor sustained adherence to very low levels of carbohydrate intake [6], all options may be considered and therapeutic choices can be determined by individual physicians and their patients

The term low-carbohydrate diet is frequently taken as synonymous with the popular Atkins diet[60] which remains highly controversial. Carbohydrate control, however, has many implementations and the severity of the epidemic of diabetes makes it appropriate to go beyond historical controversy and analyze dietary interventions as they are actually implemented.

There is reluctance to make recommendations for low carbohydrate diets on the grounds that people will not follow them but compliance and efficacy of dietary recommendations are separate phenomena. In fact, all recommendations are specifically intended to be different from average consumption[1] and it is sensibly the purpose of health agencies to encourage conformance to the best therapies.

It is time to re-appraise the role of carbohydrate restriction. Although pessimism exists in the medical community on the efficacy of any diet in the treatment of diabetes 2 and MetS, the success of carbohydrate restriction for many practitioners and individual patients[64] mandates that we should determine how this approach can be consistently and effectively employed.

Finally, while no systematic study of clinical practice has been done, anecdotal evidence suggests that carbohydrate restriction is a common clinical recommendation for diabetes. We believe that there is a need to codify these recommendations in light of current evidence.

Basic biochemistry, clinical experience and an evolving understanding of metabolic syndrome support the need for evaluation of the efficacy and safety of carbohydrate-restricted diets for the treatment of type 2 diabetes. The fact that carbohydrate restriction improves markers of cardiovascular health, even in the absence of weight loss, sensibly removes historical objections to the dangers of this approach. A critical re-appraisal could form the basis for an alternative for those patients for whom current recommendations are not successful.

## Competing interests

MCV has been a consultant for Atkins Nutritionals, Inc. All other authors declare that they have nothing to declare.

## Authors' contributions

RDF wrote the first version of this article based on published material and correspondence with the other authors who modified and approved the final version.

## Abbreviations

apo B, apolipoprotein B; apo A1, apolipoprotein A1: CVD, cardiovascular disease; HbA1c, hemoglobin A1c; HDL, high density lipoprotein; LDL, low density lipoprotein; MetS, metabolic syndrome; SFA, saturated fatty acids; TG, triglycerides (triacylglycerol); VLCKD (VLCK diet), very low carbohydrate ketogenic diet.   
